# Social Anxiety, Risk Perception, and Problematic Use of Mobile Phones and Video Games: A Gender Perspective

**DOI:** 10.3390/healthcare13222831

**Published:** 2025-11-07

**Authors:** Rosario Ruiz-Olivares, Valentina Lucena Jurado, Antonio Ruiz-García, Antonio Félix Raya Trenas

**Affiliations:** Department of Psychology, University of Cordoba, Avda. San Alberto Magno s/n, 14071 Cordoba, Spain; rosario.ruiz@uco.es (R.R.-O.); ed1lujuv@uco.es (V.L.J.); aruiz1@uco.es (A.R.-G.)

**Keywords:** video games, mobile phone, adolescents, social anxiety, risk perception, gender

## Abstract

**Background:** Adolescents spend much of their leisure time using technological devices, especially mobile phones and video games, making their use susceptible to becoming problematic. **Objectives:** This study aims to examine the relationship between social anxiety and risk perception in problematic mobile phone use (PMU) and problematic video game use (PVGU) among adolescents aged 10 to 16, considering potential gender differences. **Methods:** A total of 757 participants completed the Spanish version of the Cuestionario de uso problemático de nuevas tecnologías (UPNT), the Mobile Phone Problem Use Scale for Adolescents (MPPUSA), and the Social Anxiety Scale for Children-Revised (SASC-R). **Results:** Boys reported higher PVGU scores, and significant differences were observed between genders in perceptions of risks associated with both PMU and PVGU. No differences emerged in overall social anxiety or its subfactors. Within each gender, positive associations were observed between PMU, PVGU, and social anxiety. Predictive models explained up to 63% of the variance, identifying different risk and protective factors for boys and girls. **Conclusions:** Both PMU and PVGU should be analyzed from a gender perspective. Although there are similarities between genders, relevant differences highlight the need for tailored preventive strategies.

## 1. Introduction

Over the last decade, the use of information and communication technology (ICT) has expanded rapidly, becoming a fundamental component of the social, academic, and personal lives of adolescents worldwide [1]. The age of first exposure to devices such as smartphones and video games is steadily decreasing, often beginning in childhood [2,3]. In addition to their obvious usefulness for communication and leisure, these devices have introduced new ways of interacting, particularly through social networks and online video games. The use of technology has benefits, including improvements in hand-eye coordination, reaction time, problem-solving, spatial skills, teamwork, communication, and creativity [4,5,6,7]. However, excessive use has also raised public health concerns [1,3,4,5,6,7,8]. These dynamics take place in a complex context [2,9] marked on the one hand by social pressures—since peers and friends have and use these devices—and on the other by adults who are often permissive or approve of minors’ use of smartphones and online or offline games, failing to establish firm limits on their children’s behavior.

To date, research on the impact of technology on mental health has focused on usage time, arguing that more hours of use would lead to greater negative effects. However, more recent studies suggest that usage time is a limited predictor, providing little information about well-being or mental health [7]. Within this context, problematic technology use is defined as a behavioral pattern that causes clinically significant impairment, affecting key areas of life such as academic performance, sleep quality, and social relationships [1,8,10]. Based on this concept of problematic use, Internet gaming disorder (IGD) has been defined as a persistent pattern of video gaming that disrupts daily life, accompanied by symptoms such as preoccupation with gaming, withdrawal, and diminished or lack of interest in other activities [11,12]. Likewise, problematic mobile phone use has been described as psychological dependence and compulsive use of smartphones, which can cause anxiety and distress when the device is not available for use [13].

Some of the risk factors that have been associated with the problematic use of these devices include anxiety and impulsivity [10], the tendency to avoid or escape negative feelings, and the pursuit of or need to intensify feelings of well-being [7,12]. These data are further supported by recent longitudinal reviews showing that screen use is bidirectionally associated with socio-emotional difficulties in childhood, with more intense effects in the case of gaming [14,15]. A cross-sectional study of Canadian adolescents showed that the effects of digital use depend on the type of activity. More specifically, social media was found to be linked to internalizing symptoms, whereas video games were associated with lower loneliness and nervousness, with differences by age and gender [16]. Among the mental health factors associated with problematic technology use in adolescents, social anxiety has been shown to be particularly significant [11,12,16,17].

Social anxiety is characterized by an intense fear of social situations in which the person fears being negatively evaluated by others, which can lead to avoidance behaviors [18,19]. In contrast, online interactions may provide adolescents with a perceived sense of security where they can control how they present themselves and reduce or avoid the fear of judgment by others [12]. In a study of Chinese adolescents, Li et al. [16] found a strong association between social anxiety and problematic mobile phone use, with symptoms such as concern about negative evaluation and compulsive mobile phone use serving as the link between the two constructs. Similarly, a systematic review reported that individuals (children, adolescents, young adults, and adults) with social anxiety are at greater risk of developing problematic or addictive online gaming behaviors [11]. One explanation for this relationship is that a preference for online social interactions may mediate the relationship between social anxiety and IGD, as gamers suffering from social anxiety tend to prefer digital over face-to-face interactions [12].

Similarly, maladaptive or negative cognitions and the use of avoidance as a coping strategy have been identified as central factors in these situations [17,19]. Taken together, these studies suggest that technology use is not merely a consequence of social anxiety but rather can act as a reinforcer of the problem. Through negative reinforcement, it sustains avoidance of feared social situations and hinders the development of social skills in natural contexts.

Another relevant factor associated with problematic technology use is risk perception, defined as individuals’ subjective assessment of the likelihood of a negative event and the severity of its consequences [9]. In the context of technology use, risk perception plays a central role in moderating adolescents’ behavior. For instance, when young people underestimate the risks associated with excessive mobile phone or video game use, they may be more likely to use them in a problematic manner [20]. Ruiz-Olivares et al. [20] found that risk perception varies according to age and patterns of use. Thus, a lack of awareness of these risks (i.e., impaired academic performance, reduced sleep quality, or strained family relationships) may be a vulnerability factor for developing addictive behaviors [8]. Analyzing and understanding risk perception is essential for early detection and the design of programs to prevent problematic behaviors. Such programs can serve to identify potential dangers while addressing attitudes, beliefs, and maladaptive behaviors in technology use [3,8,20].

The impact of technology use on mental health varies separately for gender. Evidence has shown that girls are more likely to experience the negative effects of social media [3], whereas boys show a higher prevalence of problematic video game use [14]. Recent research has found that women tend to experience greater social anxiety and are more likely to use smartphones to maintain their social relationships [8,21]. Although total usage time is similar for both men and women, the reasons for use and associated psychological implications differ [1]. In this regard, a longitudinal study reported that boys and girls used smartphones differently, for example, to reduce emotional distress [22]. Conversely, a study of children and adolescents with ADHD found no gender differences in Internet, video games, or smartphone addiction [1]. However, preference for online social interactions and the presence of social anxiety have been identified as significant predictors of IGD [12].

Amid growing concerns about adolescents’ technology use, it is important to examine how social anxiety and risk perception influence problematic smartphone and video game use. The limited scientific literature on this subject highlights the need for further research. This study addresses that gap, exploring the relationship between problematic mobile phone and video game use, social anxiety, and risk perception in adolescents aged 10 to 16 years. It also undertakes gender-differential analysis to develop predictive models that incorporate the above variables.

## 2. Method

### 2.1. Participants

To determine the sample size, a simulation was conducted using the GPower 3.1 application. With an anticipated frequency of 5% and a margin of error of 0.05, it was established that a minimum of 300 individuals should be included. Following a random sampling, information was collected from 757 adolescents (32 missing cases) aged 10 to 16 in southern Spain using incidental sampling. Of these, 51.6% (374) were males and 48.4% (351) were females. The mean age of the participants was 12.15 years (SD = 1.858). Of these, 21.2% were 10 years old; 23.9% were 11; 17.9% were 12; 12.5% were 13; 9.9% were 14; 9.1% were 15; and 5.6% were 16 years old.

### 2.2. Instruments

A questionnaire was designed using the instruments listed below.

1. An ad hoc sociodemographic questionnaire was administered to collect information on the participants’ age, hours of mobile phone and video game use, age at first mobile phone use, and gender.
2. Cuestionario de uso problemático de nuevas tecnologías (UPNT) [Questionnaire on problematic use of new technologies] [23]. This questionnaire was administered to assess video game use on a 4-point Likert scale ranging from 0 (never) to 3 (always) and demonstrates acceptable reliability (Cronbach’s *α* 0.75). In our research, we have obtained a reliability index of Cronbach’s *α* 0.789.
3. Mobile Phone Problem Use Scale in Adolescents (MPPUSA). To assess problematic mobile phone use, the Spanish adaptation of the questionnaire [24] was used. The MPPUSA consists of 27 items measured on a 10-point Likert scale ranging from 1 (never) to 10 (always). To determine types of mobile phone users, the following cut-off points were established: scores from 0 to 35 indicate occasional users, 36 to 173 regular users, 174 to 181 at-risk users, and 182 to 270 problematic users [24]. The scale shows excellent reliability (Cronbach’s *α* 0.97). In the same way as in our research (Cronbach’s *α* 0.901).
4. Escala de Ansiedad Social para niños–Revised (SASC–R) [25] to assess social anxiety. The Spanish version of the scale consists of 18 items, with response options ranging from 1 (never) to 3 (often). The items are structured into three dimensions: Fear of Negative Evaluation (FNE) (items 2, 4, 6, 7, 9, 11, 13, and 14), Social Avoidance and Distress—Specific to New Peers (SAANP) (items 1, 3, 5, 8, 10, and 16), and Social Avoidance and Distress—General (SAGA) (items 12, 15, 17, and 18). The scale shows good reliability (Cronbach’s α 0.88). This date is consistent with our sample (Cronbach’s α of 0.872). Regarding the subscales, we obtained Cronbach’s α= 0.833 for the FNE, 0.730 for the SAANP, and 0.652 for the SAGA.
5. Escala de percepción del riesgo para el uso de la tecnología en niños y adolescentes (EPRUT) [21]. This scale assesses the extent to which adolescents perceive certain risks related to mobile phone, video game, and Internet use. Each item refers to a risk, such as going to bed later than you should; difficulty falling asleep; not finishing meals because of technology use; suffering violence such as fights or bullying; having less time for leisure activities such as sports, family and personal relationships; arguing with your parents; going out less with friends and receiving bad grades or not doing homework. The risks are the same for both mobile phone and video game use. EPRUT is measured on a 16-item (8 for mobile phone and 8 for videogames). Likert scale ranging from 0 (not at all) to 5 (a lot). The reliability coefficient of the EPRUT is 0.84 (Cronbach’s alpha). In our research, we obtained a Cronbach’s α of 0.816 for mobile phone use and 0.873 for video game use.

### 2.3. Procedure

Using an ex post facto cross-sectional research design, data were collected from several schools in a region in southern Spain. More than 15 primary and/or secondary schools were contacted (6000 potential participants), but only three agreed to participate in the research. One was a primary school with only two secondary school groups, and the other two were secondary-only schools. Before beginning data collection, the necessary permissions were obtained from both the schools and the families. Informed consent, child assent, and the importance of taking part in the study were explained to the participants, ensuring adherence to ethical aspects such as voluntariness, confidentiality, and anonymity, in accordance with the Declaration of Helsinki and Organic Law 3/2018 of 5 December on the Protection of Personal Data and the Guarantee of Digital Rights. The instrument described above was distributed and completed in the presence of the authors, who sought to control extraneous variables such as environmental conditions by responding to questions that arose during data collection and explaining how to complete the questionnaire. Data collection was carried out in the classroom. The average time to complete the questionnaire was 35 min. There was no material or financial compensation for participating in the study.

### 2.4. Data Analysis

Using a database in SPSS (version 25), frequency analyses were conducted to describe the number of hours spent using mobile phones and video games. Analysis of variance (ANOVA) and correlation analyses based on gender were performed to observe possible differences across variables (i.e., problematic use of mobile phones and video games, social anxiety and its subfactors, and perceptions of risks associated with mobile phones and video games). Finally, multiple linear regressions were performed, with problematic mobile phone and video game use as the dependent variables and age, perception of risk, and social anxiety as independent variables, separately for gender. Missing cases have not been considered in the analysis.

## 3. Results

As a preliminary step, we considered analyzing the hours that boys and girls spend each day using mobile phones and playing video games. Table 1 shows the percentages of boys, girls, and total separately for hours of use of both technologies. The differences observed between boys and girls are statistically significant for the use of video games, with *Χ*^2^ = 42.993 (*p* < 0.001). In the case of mobile phone use, no significant differences were found with *Χ*^2^ = 12.017 (*p* = 0.062). Regarding the age at which the participants received their first mobile phone, 69.1% had one before the age of 10, while 30.9% had one after the age of 10. Considering the cut-off points proposed by the MPPUSA, the sample is classified for boys (32% occasional, 62.8% regular, 1.9% at risk, and 3.3% problematic) and for girls (29.1% occasional, 67% regular, 1.5% at risk, and 2.4% problematic).

**Figure 1:**
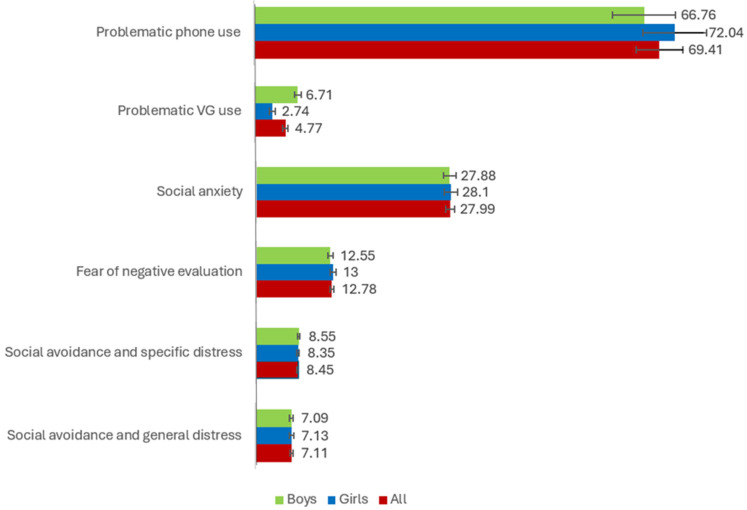
Average scores for boys, girls, and all, for PMU, PVGU, social anxiety, and its factors.

**Figure 2:**
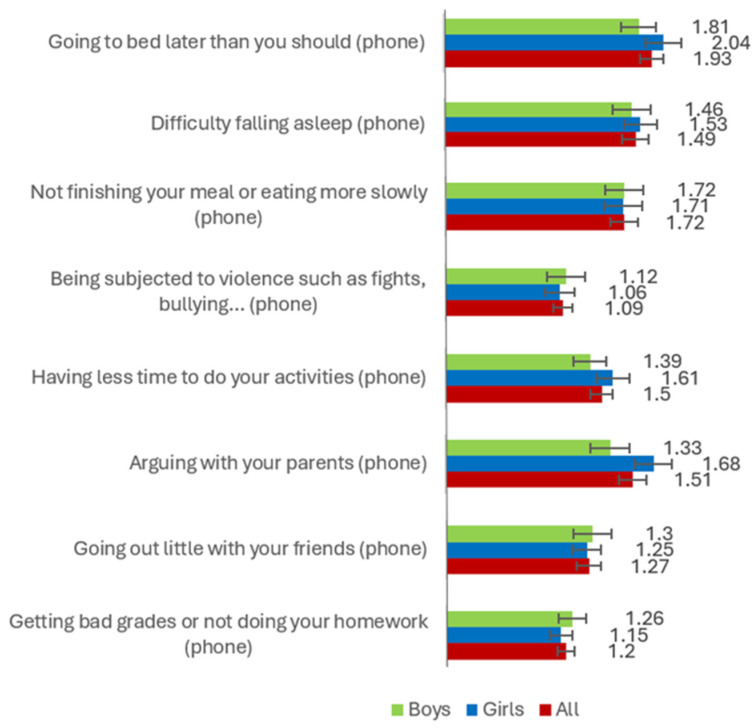
Average scores for boys, girls, and all, for risk perception related to PMU.

**Figure 3:**
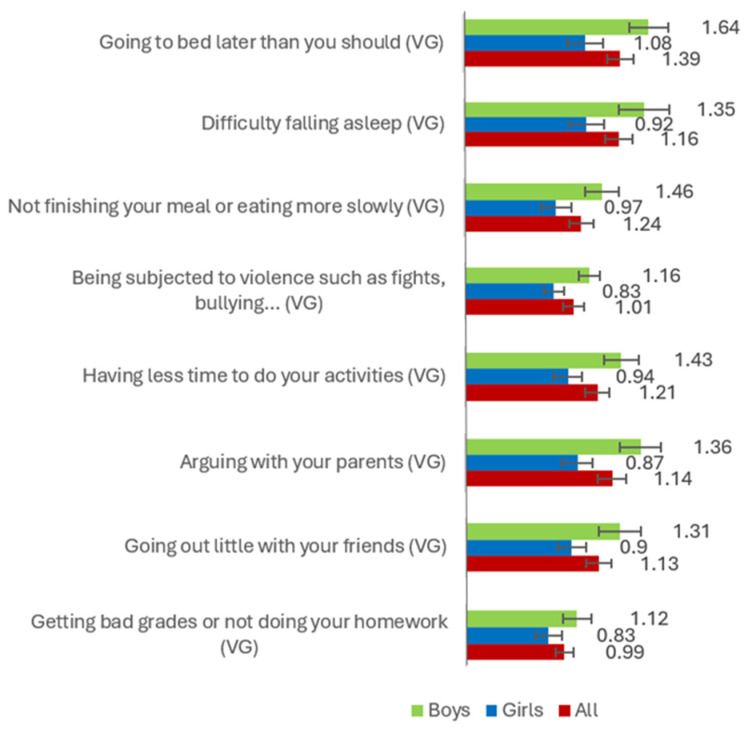
Average scores for boys, girls, and all, for risk perception related to PVGU.

Regarding gender differences in the variables considered in the study (i.e., PMU and PVGU, social anxiety and its subfactors, perception of risks associated with mobile phones and video games), Figure 1, Figure 2 and Figure 3 show the average scores obtained by boys, girls, and total participants. These mean scores show significant differences in aspects such as PVGU [*F* = 125.431 (*p* < 0.001), *µ*^2^ = 0.152], arguing with your parents (mobile phones) [*F* = 9.475 (*p* < 0.01), *µ*^2^ = 0.015], going to bed later than you should (video game) [*F* = 22.335 (*p* < 0.001), *µ*^2^ = 0.039], difficulty falling asleep (video game) [*F* = 16.683 (*p* < 0.001), *µ*^2^ = 0.030], not finishing your meal or eating more slowly because you are using these technologies (video game) [*F* = 15.521 (*p* < 0.001), *µ*^2^ = 0.027], being subjected to violence such as fights, harassment, insults, etc. (video game) [*F* = 12.085 (*p* < 0.001), *µ*^2^ = 0.021], having less time for leisure activities such as sports (video game) [*F* = 19.672 (*p* < 0.001), *µ*^2^ = 0.035], arguing with your parents (video game) [*F* = 20.209 (*p* < 0.001), *µ*^2^ = 0.035], going out less with your friends (video game) [*F* = 12.623 (*p* < 0.001), *µ*^2^ = 0.022], receiving bad grades or not doing your homework (video game) [*F* = 7.999 (*p* < 0.01), *µ*^2^ = 0.014].

Table 2 shows the relationship between the variables studied and PMU and PVGU in boys and girls. As shown in the table, usage among boys correlates positively with variables such as PVGU, age, social anxiety, and two of its subfactors (fear of negative evaluation and social avoidance and specific distress), and with all the items referring to the perception of risk in mobile phone use. PMU in girls correlates with the same variables as in boys.

On the other hand, PVGU in boys correlates with PMU, social anxiety, and two of its subfactors (fear of negative evaluation and social avoidance and specific distress), and with items regarding the perception of risk in video game use. PVGU among girls correlates with PMU, age, social anxiety, and two subfactors (fear of negative evaluation and social avoidance and general distress), as well as all the items regarding the perception of risk of video game use, except for being subjected to violence such as fights and harassment.

With the aim of designing predictive models for PMU and PVGU in a differentiated manner for boys and girls, multiple regression analyses were performed using the stepwise method. The dependent variables were PMU in boys and girls, and PVGU also in boys and girls. All other variables considered in the study (age, perception of mobile phone risk, and social anxiety) were used as predictive factors. As can be seen in Table 3, Table 4, Table 5 and Table 6, high *R*^2^ values were obtained, especially regarding mobile phone use, with variance prediction percentages of up to 63%. The fit of the models is quite adequate, with fairly moderate levels of collinearity and Durbin-Watson statistical values close to 2.

As concerns PMU among boys, Table 3 shows how PVGU, social anxiety, perceived difficulty falling asleep, and not finishing meals act as risk factors. These variables correlate positively; a higher score in one implies a higher score in the other. While social avoidance, general distress, and going out less with friends act as protective factors. That is, there is a negative correlation between them; a higher score in one implies a lower score in the other. As for PMU among girls, Table 4 shows that the variables PVGU, social anxiety, social avoidance, and specific distress, going to bed later, arguing with parents, and receiving bad grades act as risk factors. In this case, going out less with friends acts as a protective factor.

Regarding PVGU among boys, variables such as PMU, fear of negative evaluation, going to bed later, and receiving poor grades act as risk factors, while being a victim of violence or not finishing meals are protective factors (Table 5). As for girls’ problematic use of video games, going to bed later, going out little with friends, arguing with parents, social avoidance, and general distress are risk factors, while being a victim of violence and receiving poor grades act as protective factors (Table 6).

## 4. Discussion

This study examined the relationship between PMU and PVGU and social anxiety and risk perception in adolescents aged 10 to 16 years old and performed a differential analysis based on gender. In general, higher PVGU was observed in boys, while PMU was distributed more evenly between both genders. These findings are consistent with recent research that has described differential patterns of technology use depending on gender. Specifically, boys show a higher risk for video game use (*d* = 0.86), while girls exhibit a higher prevalence of mobile phone use, particularly social media (*d* = 0.67) [1,2]. These findings are especially relevant when considered in the context of recent public health data: the WHO has warned of a progressive increase in problematic use of social media and video games among European adolescents, with a higher prevalence among girls [7].

As regards social anxiety, although no significant differences were identified between genders in the overall scores or in the subfactors, a consistent association with both PMU and PVGU was found in both groups. This result is in line with studies that have identified anxiety as a robust predictor of problematic technology use in adolescents [3,4]. Cao et al. found that anxiety directly influences problematic smartphone use, with school adjustment having a mediating role and physical activity a moderating effect [5]. As for risk perception, the results show significant differences between boys and girls, thus contributing to previous evidence on the modulating role of this variable in the adoption of problematic behaviors [6].

One of the major innovations of this study is the development of gender-based predictive models with high percentages of explained variance (up to 63%), which reinforces the strength of the relationship between the variables studied. For boys, PVGU, social anxiety, and the perception of certain problems related to sleep and eating are found to be risk factors, while social avoidance and general distress are protective factors. In girls, on the other hand, risk factors include both the academic (poor school performance) and family context (conflicts with parents). This is consistent with the literature on the relevance of the social environment in shaping problematic technology use in adolescent girls [8,13].

Overall, these findings highlight the need for gender-differentiated preventive interventions that address both emotional and contextual factors. Furthermore, recent studies warn that it is not only the amount of time spent using technology that is associated with mental health problems, but especially the compulsive and addictive nature of technology use: adolescents with addictive patterns are up to three times more at risk of emotional problems [3]. This underscores the urgency of addressing the phenomenon from a multifactorial perspective by considering both social anxiety and risk perception.

### 4.1. Practical Implications

The findings of this study have important implications for the design of prevention and intervention programs in educational and community settings. Identifying risk and protective factors differentiated by gender would allow for the development of more specific and effective strategies. In the case of males/boys, it is important to consider controlling the time spent playing video games and managing social anxiety, while in females/girls, it is crucial to address the family and academic spheres by reducing conflict and promoting coping skills. Incorporating risk perception as a key variable can improve the effectiveness of awareness campaigns and digital literacy programs.

### 4.2. Limitations

This study has several limitations. First, the social desirability bias inherent in self-report: people tend to respond to what is expected of them rather than what happens. To reduce this limitation, future work is recommended using observational behavioral assessment measures, such as usage records. Furthermore, other limitations must be considered with self-report instruments, such as a lack of self-knowledge or motivation to respond. Second, the sample comes from three schools and secondary schools in a specific region in southern Spain, which may limit the generalization of the results to other regions. Another limitation is the use of a cross-sectional design, which made it difficult to establish causal relationships between the variables studied. The widespread social normalization of technological device use among adolescents and adults may also have diminished the perception of associated risks and influenced the responses obtained. Another limitation to consider is the use of multiple regression analysis, differentiated by sex, rather than an alternative analytical approach. This could increase the type I error. It would also have been interesting to provide psychometric validation data for the measures used, which would have reinforced our results. This is an objective to consider in future work.

### 4.3. Future Lines of Research

Longitudinal studies are recommended to examine the development of problematic mobile phone and video game use during adolescence, as well as their interaction with social anxiety and risk perception. It would also be interesting to incorporate mixed methods approaches combining self-reports with objective measures (e.g., time spent using applications or video games, digital records). Expanding the sample to include diverse cultural and socioeconomic contexts could help clarify the influence of these variables in predictive models. Finally, future research should explore in depth the role of family, school, and peer groups in shaping patterns of problematic use.

## 5. Conclusions

This study has investigated the relationship between problematic mobile phone and video game use, social anxiety, and risk perception in adolescents, revealing significant differences based on gender. Specific risk and protective factors have been identified, providing a more accurate framework for understanding how to address these behaviors. These results reinforce the need for gender-differentiated preventive interventions and support the inclusion of emotional and contextual variables in the design of educational and public health programs aimed at reducing the impact of problematic mobile phone and video game use in adolescence.

## Figures and Tables

**Table 1:** Percentage of boys, girls, and total according to the number of hours spent using mobile phones and playing video games.

	Mobile Phone			Video Games		
Hours	Boys	Girls	All	Boys	Girls	All
0	2.3	1.7	1.9	1.4	13.7	7
1	34.8	29.1	31.4	35	33.7	33.6
2	20.4	17	18.3	19.3	17.6	18
3	16.7	14.7	15.8	23.7	16.1	19.9
4	9.1	13.3	11	6.9	6.3	6.9
5	5.4	6.1	5.8	3.3	3.2	3.9
6 or more	11.3	18.2	15.8	10.5	8.4	10.7

**Table 2:** Pearson correlation between PMU, PVGU, and the variables considered in the study in boys, girls, and all.

	PMU		
	Boys *n* = 331	Girls *n* = 333	All *n* = 364
Problematic VG use	0.621 ** (0.550, 0.684)	0.156 ** (0.048, 0.260)	0.383 ** (0.317, 0.446)
Age	0.232 ** (0.126, 0.332)	0.402 ** (0.308, 0.489)	0.315 ** (0.245, 0.382)
Social anxiety	0.370 ** (0.245, 0.483)	0.388 ** (0.273, 0.492)	0.386 ** (0.305, 0.462)
Fear of negative evaluation	0.416 ** (0.303, 0.517)	0.353 ** (0.240, 0.457)	0.389 ** (0.312, 0.461)
Social avoidance and specific distress	0.293 ** (0.173, 0.404)	0.449 ** (0.345, 0.543)	0.381 ** (0.304, 0.453)
Social avoidance and general distress	−0.006 (−0.132, 0.121)	−0.048 (−0.169, 0.075)	−0.015 (−0.102, 0.071)
Going to bed later than you should (phone)	0.514 ** (0.424, 0.593)	0.599 ** (0.523, 0.666)	0.568 ** (0.512, 0.619)
Difficulty falling asleep (phone)	0.444 ** (0.346, 0.532)	0.480 ** (0.390, 0.561)	0.471 ** (0.407, 0.530)
Not finishing your meal or eating more slowly… (phone)	0.435 ** (0.337, 0.524)	0.316 ** (0.213, 0.413)	0.388 ** (0.319, 0.453)
Being subjected to violence such as fights, bullying (phone)	0.364 ** (0.260, 0.461)	0.141 * (0.030, 0.249)	0.281 ** (0.206, 0.352)
Having less time to do your activities… (phone)	0.341 ** (0.234, 0.439)	0.365 ** (0.265, 0.458)	0.357 ** (0.286, 0.424)
Arguing with your parents (phone)	0.356 ** (0.251, 0.454)	0.537 ** (0.453, 0.611)	0.454 ** (0.389, 0.515)
Going out little with your friends (phone)	0.275 ** (0.164, 0.379)	0.177 ** (0.067, 0.283)	0.213 ** (0.136, 0.288)
Receiving bad grades or not doing your homework… (phone)	0.361 ** (0.256, 0.457)	0.439 ** (0.345, 0.525)	0.400 ** (0.331, 0.465)
	PVGU		
	Boys *n* = 360	Girls *n* = 344	All *n* = 704
Problematic phone use	0.621 ** (0.550, 0.684)	0.156 ** (0.048, 0.260)	0.383 ** (0.317, 0.446)
Age	0.011 (−0.093, 0.115)	−0.212 ** (−0.311, −0.109)	−0.091 * (−0.164, −0.018)
Social anxiety	0.341 ** (0.221, 0.451)	0.173 ** (0.048, 0.293)	0.244 ** (0.158, 0.326)
Fear of negative evaluation	0.353 ** (0.240, 0.457)	0.123 * (0.002, 0.241)	0.199 ** (0.117, 0.280)
Social avoidance and specific distress	0.214 ** (0.096, 0.325)	−0.018 (−0.138, 0.104)	0.125 ** (0.042, 0.206)
Social avoidance and general distress	0.058 (−0.063, 0.177)	0.227 ** (0.111, 0.338)	0.138 ** (0.056, 0.219)
Going to bed later than you should (VG)	0.450 ** (0.356, 0.563)	0.377 ** (0.264, 0.479)	0.456 ** (0.388, 0.519)
Difficulty falling asleep (VG)	0.431 ** (0.334, 0.519)	0.299 ** (0.180, 0.409)	0.451 ** (0.382, 0.515)
Not finishing your meal or eating more slowly… (VG)	0.227 ** (0.117, 0.331)	0.220 ** (0.097, 0.336)	0.258 ** (0.179, 0.334)
Being subjected to violence such as fights, bullying. (VG)	0.273 ** (0.165, 0.375)	0.080 (−0.045, 0.203)	0.265 ** (0.186, 0.341)
Having less time to do your activities… (VG)	0.311 ** (0.205, 0.410)	0.257 ** (0.136, 0.370)	0.338 ** (0.263, 0.410)
Arguing with your parents (VG)	0.319 ** (0.214, 0.417)	0.343 ** (0.228, 0.449)	0.352 ** (0.277, 0.422)
Going out little with your friends (VG)	0.255 ** (0.145, 0.358)	0.362 ** (0.249, 0.466)	0.309 ** (0.231, 0.382)
Receiving bad grades or not doing your homework… (VG)	0.292 ** (0.184, 0.392)	0.234 ** (0.112, 0.349)	0.311 ** (0.234, 0.384)

* *p* < 0.05; ** *p* < 0.01.

**Table 3:** Regression model for PMU in boys.

PMU (Boys)									
	Unstandardized Coefficients		Standardized Coefficients			95% CI		Collinearity	
	*B*	*SE*	*Beta*	*t*	*p*	Low	High	Tol.	VIF
Constant	−11.265	9.022		−1.249	0.214	−29.083	6.554		
Problematic VG use	6.664	0.520	0.402	7.050	<0.001	2.637	4.690	0.803	1.245
Not finishing your meal or eating more slowly… (phone)	7.594	1.625	0.286	4.673	<0.001	4.384	10.803	0.699	1.432
Social anxiety	1.911	0.406	0.316	4.711	<0.001	1.110	2.712	0.582	1.720
Difficulty falling asleep (phone)	5.364	1.762	0.179	3.045	<0.01	1.884	8.843	0.753	1.327
Social avoidance and general distress	−2.371	0.851	−0.168	−2.785	<0.01	−4.052	−0.690	0.716	1.396
Going out little with your friends (phone)	−2.765	1.419	−0.112	−1.948	0.053	−5.567	0.038	0.794	1.260

*R*^2^ = 0.587; Adjusted *R*^2^ = 0.571; *F* = 37.414 (*p* < 0.001); Durbin-Watson = 1.890.

**Table 4:** Regression model for PMU in girls.

PMU (Girls)									
	Unstandardized Coefficients		Standardized Coefficients			95% CI		Collinearity	
	*B*	*SE*	*Beta*	*t*	*p*	Low	High	Tol.	VIF
Constant	−19.428	8.258		−2.353	0.020	−35.713	−3.143		
Going to bed later than you should (phone)	10.164	2.031	0.316	5.004	<0.001	6.158	14.170	0.471	2.125
Social avoidance and specific distress	2.611	0.852	0.184	3.063	<0.01	0.930	4.292	0.519	1.926
Receiving bad grades or not doing your homework (phone)	11.873	2.659	0.231	4.465	<0.001	6.629	17.116	0.702	1.424
Arguing with your parents (phone)	7.324	2.171	0.211	3.374	<0.001	3.043	11.604	0.481	2.080
Problematic VG use	1.870	0.588	0.146	3.182	<0.01	0.711	3.029	0.897	1.115
Going out little with your friends (phone)	−7.633	1.998	−0.177	−3.820	<0.001	−11.574	−3.692	0.877	1.140
Social anxiety	1.084	0.381	0.168	2.848	<0.01	0.333	1.835	0.538	1.858

*R*^2^ = 0.630; Adjusted *R*^2^ = 0.616; *F* = 47.846 (*p* < 0.001); Durbin-Watson = 1.884.

**Table 5:** Regression model for PVGU in boys.

PVGU (Boys)									
	Unstandardized Coefficients		Standardized Coefficients			95% CI		Collinearity	
	*B*	*SE*	*Beta*	*t*	*p*	Low	High	Tol.	VIF
Constant	−1.165	0.929		−1.255	0.212	−3.000	0.670		
Problematic phone use	0.060	0.007	0.551	8.125	<0.001	0.046	0.075	0.652	1.533
Receiving bad grades or not doing your homework… (VG)	0.877	0.307	0.192	2.858	<0.01	0.271	1.484	0.667	1.499
Fear of negative evaluation	0.221	0.079	0.179	2.810	<0.01	0.066	0.377	0.738	1.355
Difficulty falling asleep (VG)	0.901	0.296	0.232	3.042	<0.01	0.316	1.486	0.514	1.945
Being subjected to violence such as fights, bullying. (VG)	−0.601	0.300	−0.147	−2.006	<0.05	−1.193	−0.009	0.560	1.785
Not finishing your meal or eating more slowly… (VG)	−0.394	0.222	−0.115	−1.777	0.078	−0.831	0.044	0.712	1.404

*R*^2^ = 0.556; Adjusted *R*^2^ = 0.538; *F* = 30.848 (*p* < 0.001); Durbin-Watson = 1.969.

**Table 6:** Regression model for PVGU in girls.

PVGU (Girls)									
	Unstandardized Coefficients		Standardized Coefficients			95% CI		Collinearity	
	*B*	*SE*	*Beta*	*t*	*p*	Low	High	Tol.	VIF
Constant	1.096	0.873		1.255	0.212	−0.630	2.823		
Arguing with your parents (VG)	0.839	0.642	0.185	1.307	0.193	−0.431	2.109	0.260	3.841
Receiving bad grades or not doing your homework… (VG)	−2.073	0.639	−0.338	−3.245	<0.001	−3.337	−0.810	0.481	2.077
Going out little with your friends (VG)	1.873	0.579	0.359	3.236	<0.01	0.729	3.017	0.423	2.363
Social avoidance and general distress	0.163	0.096	0.127	1.698	0.092	−0.027	0.352	0.935	1.069
Being subjected to violence such as fights, bullying. (VG)	−1.346	0.488	−0.305	−2.757	<0.01	−2.310	−0.381	0.427	2.343
Going to bed later than you should (VG)	1.046	0.507	−0.295	2.046	<0.05	0.044	2.047	0.255	3.923

*R*^2^ = 0.259; Adjusted *R*^2^ = 0.228; *F* = 8.279 (*p* < 0.001); Durbin-Watson = 2.082.

## Data Availability

The data or result files can be requested from the corresponding author.

## References

[1] Menéndez-García A., Jiménez-Arroyo A., Rodrigo-Yanguas M., Marín-Vila M., Sánchez-Sánchez F., Roman-Riechmann E., Blasco-Fontecilla H. (2022). Adicción a Internet, videojuegos y teléfonos móviles en niños y adolescentes: Un estudio de casos y controles. Adicciones.

[2] Infante C.R.M., Peláez K.F.C., Cedeño D.C., León M.E.M., Monar A.M.Q. (2025). El impacto de la tecnología en la vida social de los adolescentes. Rev. Multidiscip. Estud. Gen..

[3] Virós-Martín C., Montaña-Blasco M., Jiménez-Morales M. (2024). Can’t stop scrolling! Adolescents’ patterns of TikTok use and digital well-being self-perception. Humanit. Soc. Sci. Commun..

[4] Alanko D. (2023). The health effects of video games in children and adolescents. Pediatr. Rev..

[5] Bilali A., Katsiroumpa A., Koutelekos I., Dafogianni C., Gallos P., Moisoglou I., Galanis P. (2025). Association between TikTok use and anxiety, depression, and sleepiness among adolescents: A cross-sectional study in Greece. Pediatr. Rep..

[6] Granic I., Lobel A., Engels R.C.M.E. (2014). The benefits of playing video games. Am. Psychol..

[7] Sauter M., Braun T., Mack W. (2021). Social context and gaming motives predict mental health better than time played: An exploratory regression analysis with over 13,000 video game players. Cyberpsychol. Behav. Soc. Netw..

[8] Sharma K., Sarathamani T., Bhougal S.K., Singh H.K. (2021). Smartphone-induced behaviour: Utilisation, benefits, nomophobic behaviour and perceived risks. J. Creat. Commun..

[9] Gutiérrez Arenas M.P., García-Rojas A.D., Hernando Gómez A., Prieto Medel C. (2025). Percepción del riesgo de los menores ante el uso de las tecnologías: Influencia de diferentes variables. Rotura–Rev. Comun. Cult. Artes.

[10] Shaltout N. (2025). Investigating the Risk and Protective Factors of Internet Addiction Among Adolescents Through the Lens of Cognitive Behavioral Theory: A Cross-Sectional Study. Master’s Thesis.

[11] Gioia F., Mariano Colella G., Boursier V. (2022). Evidence on problematic online gaming and social anxiety over the past ten years: A systematic literature review. Curr. Addict. Rep..

[12] Marino C., Canale N., Vieno A., Caselli G., Scacchi L., Spada M.M. (2020). Social anxiety and internet gaming disorder: The role of motives and metacognitions. J. Behav. Addict..

[13] Wei J., Dang J., Mi Y., Zhou M. (2024). Mobile phone addiction and social anxiety among Chinese adolescents: Mediating role of interpersonal problems. An. Psicol..

[14] Vasconcellos R.P., Sanders T., Lonsdale C., Parker P., Conigrave J., Tang S., Cruz B.d.P., Biddle S.J.H., Taylor R., Innes-Hughes C. (2025). Electronic screen use and children’s socioemotional problems: A systematic review and meta-analysis of longitudinal studies. Psychol. Bull..

[15] Zhang J., Browne D., Children and Youth Planning Table of Waterloo Region (2025). Digital media use, social isolation, and mental health symptoms in Canadian youth: A psychometric network analysis. Can. J. Behav. Sci..

[16] Li S., Feng N., Cui L. (2024). Network analysis of social anxiety and problematic mobile phone use in Chinese adolescents: A longitudinal study. Addict. Behav..

[17] Zhou H., Jiang H., Zhang B., Liang H. (2021). Social anxiety, maladaptive cognition, mobile phone addiction, and perceived social support: A moderated mediation model. J. Psychol. Afr..

[18] American Psychiatric Association (2022). Manual Diagnóstico Y Estadístico de los Trastornos Mentales (DSM-5).

[19] Plessis C., Guerrien A., Altintas E. (2025). Sociotropy and video game playing: Massively multiplayer online role-playing games versus other games. Encephale.

[20] Ruiz-Olivares R., Casas J.A., Lucena V., Aguilar-Yamuza B. (2024). Propiedades psicométricas de la “Escala de percepción del riesgo del uso de la tecnología para niños y adolescentes”. Behav. Psychol. Psicol. Conductual..

[21] Haro B., Beranuy M., Vega M.A., Calvo F., Carbonell X. (2022). Uso problemático del móvil y diferencias de género en formación profesional. Educ. XX1.

[22] Xu X., Li H., Bai R., Liu Q. (2024). Do boys and girls display different levels of depression in response to mobile phone addiction? Examining the longitudinal effects of four types of mobile phone addiction. Psychol. Res. Behav. Manag..

[23] Labrador F.J., Villadangos S.M., Crespo M., Becoña E. (2013). Design and validation of the new technologies problematic use questionnaire. An. Psicol..

[24] López-Fernández O., Honrubia-Serrano M.L., Freixa-Blanxart M. (2012). Adaptación española del “Mobile Phone Problem Use Scale” para población adolescente. Adicciones.

[25] Chorot P., Valiente R.M., Santed Germán M.A., Sánchez-Arribas C. (2025). Estructura factorial de la escala de ansiedad social para niños-revisada (SASC-R). Rev. Psicopatol. Psicol. Clín..

